# Exploring patient information needs in type 2 diabetes: A cross sectional study of questions

**DOI:** 10.1371/journal.pone.0203429

**Published:** 2018-11-16

**Authors:** Colleen E. Crangle, Colin Bradley, Paul F. Carlin, Robert J. Esterhay, Roy Harper, Patricia M. Kearney, Vera J. C. McCarthy, Michael F. McTear, Eileen Savage, Mark S. Tuttle, Jonathan G. Wallace

**Affiliations:** 1 Department of Health Management and Systems Sciences, School of Public Health and Information Sciences, University of Louisville, Louisville, Kentucky, United States of America; 2 Department of General Practice, University College Cork, Cork, Ireland; 3 South Eastern Health and Social Care Trust, Research Office, Dundonald, Northern Ireland, United Kingdom; 4 Department of Endocrinology & Diabetes, The Ulster Hospital, South Eastern Health and Social Care Trust, Dundonald, Northern Ireland, United Kingdom; 5 Department of Epidemiology & Public Health, University College Cork, Cork, Ireland; 6 School of Nursing and Midwifery, University College Cork, Cork, Ireland; 7 School of Computing and Mathematics, University of Ulster at Jordanstown, Jordanstown, Northern Ireland, United Kingdom; 8 Board of Directors, Apelon Inc., Hartford, Connecticut, United States of America; Weill Cornell Medical College Qatar, QATAR

## Abstract

This study set out to analyze questions about type 2 diabetes mellitus (T2DM) from patients and the public. The aim was to better understand people’s information needs by starting with what they do not know, discovered through their own questions, rather than starting with what we know about T2DM and subsequently finding ways to communicate that information to people affected by or at risk of the disease. One hundred and sixty-four questions were collected from 120 patients attending outpatient diabetes clinics and 300 questions from 100 members of the public through the Amazon Mechanical Turk crowdsourcing platform. Twenty-three general and diabetes-specific topics and five phases of disease progression were identified; these were used to manually categorize the questions. Analyses were performed to determine which topics, if any, were significant predictors of a question’s being asked by a patient or the public, and similarly for questions from a woman or a man. Further analysis identified the individual topics that were assigned significantly more often to the crowdsourced or clinic questions. These were Causes (CI: [-0.07, -0.03], p < .001), Risk Factors ([-0.08, -0.03], p < .001), Prevention ([-0.06, -0.02], p < .001), Diagnosis ([-0.05, -0.02], p < .001), and Distribution of a Disease in a Population ([-0.05,-0.01], p = .0016) for the crowdsourced questions and Treatment ([0.03, 0.01], p = .0019), Disease Complications ([0.02, 0.07], p < .001), and Psychosocial ([0.05, 0.1], p < .001) for the clinic questions. No highly significant gender-specific topics emerged in our study, but questions about Weight were more likely to come from women and Psychosocial questions from men. There were significantly more crowdsourced questions about the time Prior to any Diagnosis ([(-0.11, -0.04], p = .0013) and significantly more clinic questions about Health Maintenance and Prevention after diagnosis ([0.07. 0.17], p < .001). A descriptive analysis pointed to the value provided by the specificity of questions, their potential to disclose emotions behind questions, and the as-yet unrecognized information needs they can reveal. Large-scale collection of questions from patients across the spectrum of T2DM progression and from the public–a significant percentage of whom are likely to be as yet undiagnosed–is expected to yield further valuable insights.

## Introduction

Diabetes is a major health problem worldwide. The prevalence of global, age-standardized diabetes is 9% in men and 7.9% in women, with the number having risen around the globe from 108 million in 1980 to 422 million in 2014 [1]. Type 2 diabetes mellitus (T2DM) is a main driver of the increase, accounting for approximately 90% of all diabetes cases [2–4]. Diabetes is a complex condition and people with diabetes have a diverse range of information needs [5–8]. Large-scale investigations such as the DAWN studies on the attitudes, wishes and needs of patients and caregivers [9, 10] have told us much, but research to date has paid little attention to exploring the information needs of patients as expressed in the questions they have about diabetes. Questions convey information needs in the patient’s own voice and permit the individual and subjective experience of illness to be captured [11]. To our knowledge, no-one to date has investigated on a large scale what T2DM patients want to know at different stages of diagnosis and treatment by asking them directly what their questions are, nor have questions from the public been solicited and examined.

Our study concerns a new way of thinking about patient information needs in diabetes, starting not with what we know about T2DM and finding ways to communicate that information to patients but starting with what patients do not know, discovered through their own questions. Soliciting, and then responding, to patient questions on a large scale has the potential to create a new information resource for T2DM, both in terms of content and organization. A questions-based approach to patient knowledge is distinct from active information seeking through which the patient searches extant information resources [5], and it is distinct from passive information receipt in which the patient is exposed either accidentally or deliberately to extant information resources [12]. A questions-based approach has the potential to create a dynamic, continually updated resource that will capture patient information needs as they evolve over time.

It is estimated that more than half of American adults have either T2DM or prediabetes (as measured by blood sugar levels or determined by diagnosis) and of those more than one-third are unaware they have the disease [13]. Consequently, it is crucial that we understand the information needs and voice of those who do not have diabetes, or do not know they have diabetes, but still have questions whether out of curiosity or concern for themselves or a loved one. In this paper, we report on the first stage of our work soliciting questions directly from both patients and the general public and analyzing the questions to see what they reveal.

Questions play a vital role in health care. Patient questions foster good communication with health professionals, resulting in better care and the right care at the right time [14–17]. However, poor bi-directional flow of information between the diabetes health professional and the patient has been documented. Discrepancies have also been noted between information provided by health care providers and what patients with diabetes need [18]. Patients often cannot get as much detail as they need during office visits [19]. Time constraints, whether actual or perceived, prevent some patients from asking questions during the consultation [5, 20]. Patients also find it difficult to retain much of what they have been told by a health professional, and what they do remember is incorrect almost half the time [21–23].

Clinical information needs have been extensively studied by collecting questions from physicians and analyzing them [24–44]. For patients and the general population, the situation is very different. Only recently have their health questions been studied in any depth [45–51], with few studies, to our knowledge, focusing on diabetes or investigating differences between questions from patients and those not in a patient setting. Our recent study has shown that available online sources of information do not provide answers to patient questions about diabetes and that there is an urgent need to better understand these information needs [52]. In this study, therefore, we set out to collect and investigate questions about diabetes from two sources, namely, patients attending a diabetes clinic and the public through crowdsourcing. We hypothesized that an analysis of the questions in terms of the topics they cover and the phases of disease progression they concern would provide important insights, potentially also revealing differences in information needs between patients and those outside the patient setting, who may or may not have diabetes or may be unaware they have the disease.

## Methods

### Ethics statement

This study makes secondary use of anonymized data. A prior service evaluation had been approved within the South Eastern Health and Social Care Trust, Northern Ireland, to assess patient information needs by approaching patients attending the diabetes clinic and asking them to provide questions. They were free to refuse if they wished to. No participant consent was needed for the service evaluation and none was sought. Questions were recorded on a sheet provided to each patient if interested, with no identifiers such as clinic time, clinician or personal information collected. No ethics committee approval was needed for our secondary analysis of the collected questions. This practice conforms to the guidelines of the Health Research Authority of the UK National Health Service and current UK legislative and good practice arrangements. The authors had no direct contact with the participants and there were no minors among the participants.

### Question collection

As part of a prior service evaluation, all patients attending the weekly diabetes outpatient clinic at the Ulster Hospital in Northern Ireland during February to April, 2014 had been invited to submit questions by responding to the following: *What are the one or two most pressing questions about your diabetes that you would like answered*? Patients were provided with a blank page to record their questions and questions from the same individual were marked as such.

We obtained additional questions using the crowdsourcing platform of Amazon Mechanical Turk (AMT). Crowdsourcing has become an important part of many clinical studies [53], with new platforms emerging to meet the particular requirements of research [54]. One hundred AMT participants were asked to each enter three questions s/he had about diabetes. Each participant was asked to specify age, sex/gender and if s/he had a diagnosis of type 2 or type 1 diabetes, or a diagnosis of diabetes but did not know the type, and if s/he had a friend or family member with a diagnosis of type 2, type 1 or unknown type. Crowdsourced question collection took place on July 8^th^ -11^th^, 2015.

The clinic and crowdsourced question corpora are given in S1 File and S2 File. All questions are presented as written by the participants, with spelling and punctuation intact.

### Categorization by topic and phase

Question content was determined through fine-grained manual categorization of the topics and the phases of diabetes progression the question referred to. Such detailed assessment of need is part of the move towards better disease management through understanding the likely information needs of different subgroups of people at different phases of the disease, at the onset of diabetes, for example, or later when a new complication has developed.

An initial set of 13 topics, based on known concerns of patients with diabetes [5–10, 55–57] and our prior work on consumer questions [16], was compiled and used to conduct a preliminary categorization of the crowdsourced questions. This undertaking led to an expanded set of 23 topics for use in this study. We additionally compiled a five-part patient-oriented classification of the phases of T2DM drawing on prior work and our clinical experience [58–67].

Two researchers independently categorized each question by topic and phase. A question could fall under more than one topic and more than one phase, but the phases had to be consecutive, as in the range 3–5, for instance. There were therefore more question-topic assignments and more question-phase assignments than there were questions.

Coding was performed by CEC (all crowdsourced questions), PK (half the crowdsourced questions), VMC (half the crowdsourced questions), and PC and RH (all clinic questions each). PK and RH are clinicians, VMC and PC healthcare researchers, and CEC a non-clinical bioinformatics researcher. For each question and topic, a score of 1 indicated that the question fell under that topic and a score of 0 that it did not. If both coders scored 1 or 0 for a question and topic, it was counted as agreement. Agreement for phase was determined by an overlap between one coder and the other. Intercoder reliability was computed using Cohen’s kappa with the following guidelines from [68]: slight agreement (0–0.2); fair (0.21–0.4); moderate (0.41–0.6); substantial (0.61–0.8); almost perfect (0.81–1). Disagreement between coders was resolved through consensus review by the coders and members of the project team.

### Statistical analysis

The following analyses were performed for topics and stratified by sex for the crowdsourced questions. The significance threshold was set at .05 except where indicated.

Because consecutive questions are more likely to stem from the same questioner in each corpus, the samples cannot be assumed to be independent. We therefore determined which, if any, individuals had highly correlated questions in terms of their topic assignments using the Pearson correlation coefficient. Then, following the guideline that multicollinearity may be a problem in a data set if any pairwise |r| > 0.7 [69], we removed the questions from any individual who had strongly correlated questions (|r| > 0.7 for any pair of his/her questions). For each corpus, we also examined all pairwise correlations between topics, in terms of the questions assigned to them, removing those topics, if any, that were strongly correlated.

To determine which topics, if any, were significant predictors of a question’s coming from a patient in the clinic or from the public through crowdsourcing, we used Lasso regression with the Least Angle Regression (LARS) algorithm [70,71], Lasso-LARS is a model selection algorithm that uses repeated internal cross-validation to select variables and estimate coefficients in the presence of collinearity. We applied Lasso-LARS both before and after removal of highly correlated questions and topics. Computations were performed using the *LassoLarsCV* function from the *scikit-learn* python package with 10-fold cross validation and default parameters [72]. Lasso-LARS regression was also performed on the crowdsourced questions to determine which, if any, topics were significant predictors of a question’s coming from a woman or a man.

We also examined each topic individually to determine if it was assigned significantly more often to the clinic or the crowdsourced questions, correcting for multiple comparisons using the Benjamini–Hochberg false discovery rate (FDR) [73–75]. For the crowdsourced questions only, we similarly asked for each topic if it was assigned significantly more often to the questions asked by men or those asked by women. The 2-tailed z-test provided 95% confidence intervals (CI) for these estimates. This analysis told us something about the topics, in contrast to the Lasso-LARS analysis that told us something about the questions and the people asking them. The z-tests were performed after confirmation that the distribution of questions over topics was approximately normal. That is, we confirmed that the number of questions per topic was approximately normally distributed for both the crowdsourced and clinic questions under the Shapiro-Wilk test, both before and after removal of the correlated questions, and similarly for the female and male questions [76]. For the phases of disease progression, a similar analysis was done to determine which phases, if any, were assigned significantly more often to the clinic or the crowdsourced questions.

To gain additional understanding of the differences between the clinic and crowdsourced questions, the top three (85th percentile) and top five (75% percentile) topics in terms of the number of questions to which they were assigned were identified for each corpus. Those that were top in one corpus and not the other were recognized as characteristic of that corpus. A similar analysis was done for the phases of disease progression.

### Descriptive analysis

In addition to topic analysis and the analysis by phase of disease progression, the combined corpus of questions was reviewed from a holistic and descriptive perspective to ascertain any inferences implicit in the questions that might reveal underlying concerns or issues for the person generating the question. It was apparent that the questioners, not all of whom had diabetes, were seeking more than just factual information. A limited qualitative analysis of the combined corpus was therefore undertaken to address this need for a broader interpretation of the questions beyond their literal content. This analysis was not exhaustive but illustrative, identifying themes that might inform a detailed analysis of a larger collection of questions.

## Results

### The topics

A preliminary categorization of the crowdsourced questions using a core set of categories derived from earlier work [5–10, 16,55–57] produced a Cohen’s kappa score of 0.61 overall, which represents moderate to substantial agreement [68]. A subsequent round-table discussion by members of the project team (CEC, PK, VMC, MFM, ES, JGW and PC) led to the formulation of the 23 categories described in Table 1. Several diabetes-specific categories, namely Lifestyle / Behavior Change (hereafter abbreviated simply to Lifestyle), Exercise, Diet, Weight, and Cure or Reversal, were added to the core categories. For this last topic, we note that a more clinically oriented topic descriptor would be Control or Remission. However, our experience to date with patient and general-public questions is that the lay perception centers on the idea of completely getting rid of a disease and for this reason we use the descriptor Cure or Reversal. The topic of Complications derived from earlier work was split into Disease Complications and Treatment Complications to properly represent the types of questions found for T2DM.

**Table 1:** Topic categories for T2DM questions.

**Causes**	Questions about the causes of diabetes or one of its complications. Includes causal factors that might increase risk and causes of symptoms.
**Risk Factors**	Questions about factors that raise the risk of developing diabetes or any of its complications (not necessarily causal factors, for example, gender).
**Prevention**	Questions about the prevention of diabetes or the prevention of complications arising from diabetes.
**Diagnosis**	Questions about diagnostic tests for diabetes or any of its complications. Includes questions about signs or symptoms that might lead to a diagnosis. Includes methods for determining the difference between pre-diabetes, type 1 and type 2.
**Manifestations**	Questions about signs or symptoms of diabetes or any of its complications.
**Treatment**	Questions about treatments for diabetes. Includes medication and self-management behaviors that could be part of a treatment plan.
**Anatomy**	Questions that make reference to any particular part of the body, such as questions about a location affected by diabetes.
**Cure / Reversal**	Questions about a cure for diabetes or about the reversal of symptoms to the point where one could be considered condition free or in remission.
**Diet / Nutrition**	Questions about the role of diet or nutrition in the prevention, development or management of diabetes and its complications.
**Exercise**	Questions about the role of exercise in the prevention, development or management of diabetes and its complications.
**Weight**	Questions about the role of weight in the prevention, development or management of diabetes and its complications.
**Lifestyle**	Questions about things a person can or must do to prevent or manage diabetes or its complications (including diet, exercise, or weight).
**Disease Complications**	Questions about the problems diabetes causes. This includes the risks faced by patients with diabetes and the nature and experience of the complications.
**Treatment Complications**	Questions about problems arising from specific treatments for diabetes or one of its complications.
**Person or Organizations**	Questions about a person or organization involved with a disease. This can include medical specialists, hospitals, research teams, insurance payments, or support groups for a particular disease.
**Prognosis**	Questions asking about life expectancy, quality of life, or the probability of success of a given treatment.
**Distribution of a Disease in a Population**	Questions about the occurrence of diabetes in a population and questions about the distribution of complications in the population of people with diabetes.
**Inheritance Patterns**	Questions about inheritance patterns in diabetes.
**Transmission Patterns**	Questions about transmission patterns for diabetes (when conceived of as an infectious disease).
**Research**	Questions about research on diabetes. Includes questions about clinical trials.
**Psychosocial**	Questions about the social-emotional ramifications of diabetes.
**Own Health Record Related**	Questions that relate specifically to the questioner’s own health or that reference information in the person’s health record. Includes questions about “my” medication, etc.
**Other**	Questions that do not belong to any of the above. Includes non-medical questions about a disease, such as policy decisions, for example.

### The questions

One hundred and sixty-four questions were collected from 120 patients during 12 outpatient clinics. Most of the questions were about diabetes (N = 155) with the remainder related to clinic operation (N = 9). Of the questions on diabetes, 152 from 101 patients were about T2DM and these questions were retained for our analysis. Although only 1 to 2 questions were asked for, 2 patients gave 3 questions each. Most of the patients attending the clinic had T2DM (95%). These questions are given in S1 File.

For the crowdsourced questions, 100 AMT participants each contributed three questions about diabetes (N = 300). Most of the questions were about T2DM (N = 284) with a smaller number related to type 1 diabetes (N = 15) and 1 question duplicated by one of the questioners. Of the 100 questioners (F 34, M 66), 9 had diabetes (6 type 2, 2 type 1, one unknown type) and 91 a friend or family member with diabetes (30 type 2, 17 type 1, 44 unknown type). The 284 questions about T2DM were retained for analysis. These questions are given in S2 File. For the clinic questions overall, agreement between the coders was substantial (Cohen’s kappa = 0.77, SD = 0.18). For the crowdsourced questions overall, agreement between the coders was almost perfect (Cohen’s kappa = 0.86 SD = 0.1). Disagreements were resolved by consensus between the coders.

We found that for the crowdsourced questions, 16 of the 100 individuals had strongly correlated questions (|r| > 0.7 for any pair of their 2 or 3 questions) and, for the clinic questions, 3 of the 101 patients. After removing the questions from the identified individuals there were 236 crowdsourced questions from 84 individuals and 147 clinic questions from 98 patients remaining. We did not find the topics in the crowdsourced questions to be correlated at the 0.7 criterion value, but for the clinic questions, Transmission Patterns was correlated with Inheritance Patterns at the 0.7 level. We therefore dropped the category Transmission Patterns, which had only 2 questions in the crowdsourced corpus and 5 in the clinic corpus, all of which also fell under other topic categories and consequently did not need to be removed.

### Topics and the clinic and crowdsourced questions

The clinic questions had an average of 2.8 topics per question (min 1, max 7) and the crowdsourced questions had an average of 2.1 topics per question (min 1, max 5). The results of the Lasso-LARS regression on all questions showed slightly higher odds ratios in favor of questions that were Own Health Record Related and about Treatment coming from the clinic patients (1.143 and 1.114 respectively). The odds ratios for all other questions were less than 1.062. The optimum alpha value found was 0.0009 with a mean squared error of 0.151 for both the training and test data. Lasso-LARS regression on only the non-correlated questions revealed similar slightly higher odds ratios in favor of the clinic questions for the same two topics (1.173 and 1.156 for Own Health Record Related and Treatment, respectively), with all other odds ratios less than 1.1. The optimum alpha value was 0.0006 with a mean squared error for the training data of 0.132 and 0.187 for the test data.

In terms of the individual topics, topics that were assigned significantly more often to the crowdsourced than the clinic questions were Causes (CI: [-0.07, -0.03], p < .001), Risk Factors ([-0.08, -0.03], p < .001), Prevention ([-0.06, -0.02], p < .001), Diagnosis ([-0.05, -0.02], p < .001), and Distribution of a Disease in a Population ([-0.05,-0.01], p = .0016). In contrast, the topics Treatment ([0.03, 0.01], p = .0019), Disease Complications ([0.02, 0.07], p < .001), and Psychosocial ([0.05, 0.1], p < .001) were assigned significantly more often to the clinic questions. See Table 2.

**Table 2:** Topics assigned to the crowdsourced and clinic questions.

	Crowdsourced (N = 236)	Clinic (N = 147)	CI*	FDR-adjusted p-values, 2-tailed z-test
**Causes**	49 / 675**	14 / 598**	(-0.07, -0.03)	< .001
**Risk Factors**	64 / 675	23 / 598	(-0.08, -0.03)	< .001
**Prevention**	33 / 675	7 / 598	(-0.06, -0.02)	< .001
**Diagnosis**	31 / 675	6 / 598	(-0.05, -0.02)	< .001
**Manifestations**	45 / 675	43/ 598	(-0.02, 0.03)	.747
**Treatment**	61 / 675	91 / 598	(0.03, 0.01)	.0019
**Anatomy**	20 / 675	29 / 598	(-0.0, 0.04)	.1383
**Cure / Reversal**	47 / 675	28 / 598	(-0.05, 0.0)	.1383
**Diet / Nutrition**	8 / 675	5 / 598	(-0.01, 0.01)	.6942
**Exercise**	12 / 675	9 / 598	(-0.02, 0.01)	.747
**Weight**	65 / 675	56 / 598	(-0.03, 0.03)	.8721
**Lifestyle**	33 / 675	43 / 598	(-0.0, 0.05)	.1383
**Disease Complications**	16 / 675	40 / 598	(0.02, 0.07)	< .001
**Treatment Complications**	7 / 675	16 / 598	(0.0, 0.03)	.0696
**Person or Organization**	41 / 675	41 / 598	(-0.02, 0.03)	.6973
**Prognosis**	18 / 675	7 / 598	(-0.03, -0.0)	.1207
**Distribution of a Disease in a Population**	26 / 675	5 / 598	(-0.05, -0.01)	.0016
**Inheritance Patterns**	15 / 675	20 / 598	(-0.01, 0.03)	.3251
**Research**	20 / 675	20 / 598	(-0.02, 0.02)	.747
**Psychosocial**	4 / 675	47 / 598	(0.05, 0.1)	< .001
**Own Health Record Related**	27 / 675	30 / 598	(-0.01, 0.03)	.5244

*Confidence intervals at the .05 level.

**The denominator in each column is the number of topic assignments in total for the corpus.

The three most frequent clinic topics (Table 2) were Treatment (91 questions), Weight (56) and Psychosocial (47). The three most frequent crowdsourced topics also included Weight (65) and Treatment (61), but included Risk (64) rather than Psychosocial. The topic Psychosocial therefore characterizes the clinic questions and the topic Risk Factors the crowdsourced questions. The next two clinic topics were Manifestations (43) and Lifestyle (43), which were not in the top crowdsourced topics, and therefore further characterize the clinic questions. The next two crowdsourced topics were Causes (49) and Cure / Reversal (47), which were not in the top clinic questions, and therefore further characterize the crowdsourced questions.

For the crowdsourced questions, Lasso-LARS regression showed a slightly higher odds ratio (1.122) for a question about Weight coming from a woman rather than a man. The optimum alpha value found was 0.0024 and the mean squared error for the training data was 0.193 and 0.235 for the test data.

In terms of the individual topics, the only topic that was more strongly associated with one gender over another was Psychosocial (CI: [0.02, 0.05], p = .1497), which was more strongly represented in the male questions, but only at the 0.2 level. For both men and women, the topics Lifestyle (women 24 questions; men 41 questions), Risk Factors (24; 41), and Diet (19; 28) were most frequently assigned. There was one topic appearing uniquely in the top five for men and one for women that might in addition be thought of as characterizing the two groups. These were Manifestations (women 18 question) and Prognosis (men 24 questions).

### The phases of disease progression

The five phases of T2DM progression we identified from the literature and our experience were: Prior to any Diagnosis; Pre-diabetic (diagnosed); Onset of T2DM; Health Maintenance and Prevention; Complications–Minor (onset) or Major (dominance). These are listed in Table 3 along with a description of each phase.

**Table 3:** Phases of disease progression for T2DM.

Phase 1	**Prior to any Diagnosis**	Given that 27.8% (8.1 million) of those estimated to have diabetes in the USA are undiagnosed, it is important to understand the questions people may have prior to a diagnosis. Given the increasing prevalence of T2DM, many people have family members or friends with a diagnosis. Questions may therefore not be about their own susceptibility but about giving support to others with the condition.
Phase 2	**Pre-diabetic (diagnosed)**	On being diagnosed as pre-diabetic, a person’s questions may reveal a response of confusion, denial or fear. In coming to terms with the fact that s/he may be facing a serious chronic illness, the person may have questions about the choices s/he has to make.
Phase 3	**Onset of T2DM**	With the onset of T2DM, questions may reveal a response of anger or denial or a wait-and-see attitude. In accepting that s/he has to live with a serious chronic illness, the person may have questions about new knowledge that must be acquired and a new and possibly demanding self-care regimen that must be adjusted to. If the onset of diabetes is abrupt, the adjustment may be particularly difficult and questions may reflect this struggle.
Phase 4	**Health Maintenance and Prevention**	After initial diagnosis, questions may reveal a person energized to manage his/her diabetes. A lack of questions may indicate a person trying to ignore his/her diabetes. As treatment focuses on the prevention of diabetes complications, questions may focus on the new self-care behaviors that must be maintained.
Phase 5	**Complications–Minor (onset) or Major (dominance)**	With the onset of complications, a person may be energized by the complications to manage his/her diabetes. Or s/he may respond with fatalism or increased distress. Questions may reflect attempts to understand and accept a new condition trajectory. Questions may focus on maximizing quality of life, especially as complications come to dominate the person’s life. Questions may seek emotional support, not just knowledge, as the person’s self-image as a functioning, healthy adult undergoes possible change. The person must learn to live with challenges that may affect his/her activity levels, functional abilities, and emotional and social well-being. As the person’s condition progresses, new questions will arise.

For both the clinic and consumer questions, intercoder reliability for categorization by phase was moderate (k = 0.64 clinic; k = 0.67 crowdsourced). Given the exploratory nature of this categorization by phase, by consensus the coders agreed to assign a phase category to a question if either one of the coders did so. In this way, the judgements of all coders (clinical and non-clinical) could be taken into account.

### Phases and the clinic and crowdsourced questions

There were significantly more crowdsourced questions that concerned Phase 1, the time Prior to any Diagnosis (CI: [-0.11, -0.04], p = .0013), and significantly more clinic questions about Phase 4, Health Maintenance and Prevention (CI: [0.07. 0.17], p < .001). See Table 4.

**Table 4:** Phases assigned to the crowdsourced and clinic questions.

	Crowdsourced (N = 236)	Clinic (N = 147)	CI*	FDR-adjusted p-values, 2-tailed z-test
**Phase 1: Prior to any Diagnosis**	118 / 756**	32 / 386**	(-0.11, -0.04)	.0013
**Phase 2: Pre-diabetic (diagnosed)**	127 / 756	49 / 386	(-0.08, 0.0)	.0692
**Phase 3: Onset of T2DM**	220 / 756	91 / 386	(-0.11, -0.0)	.0647
**Phase 4: Health Maintenance and Prevention**	148 / 756	122 / 386	(0.07, 0.17)	< .001
**Phase 5: Complications–Minor (onset) or Major (dominance)**	143 / 675	92 / 386	(-0.0, 0.1)	.0647

*Confidence intervals at the .05 level.

**The denominator in each column is the number of phase assignments in total for the corpus.

**Fig 1:**
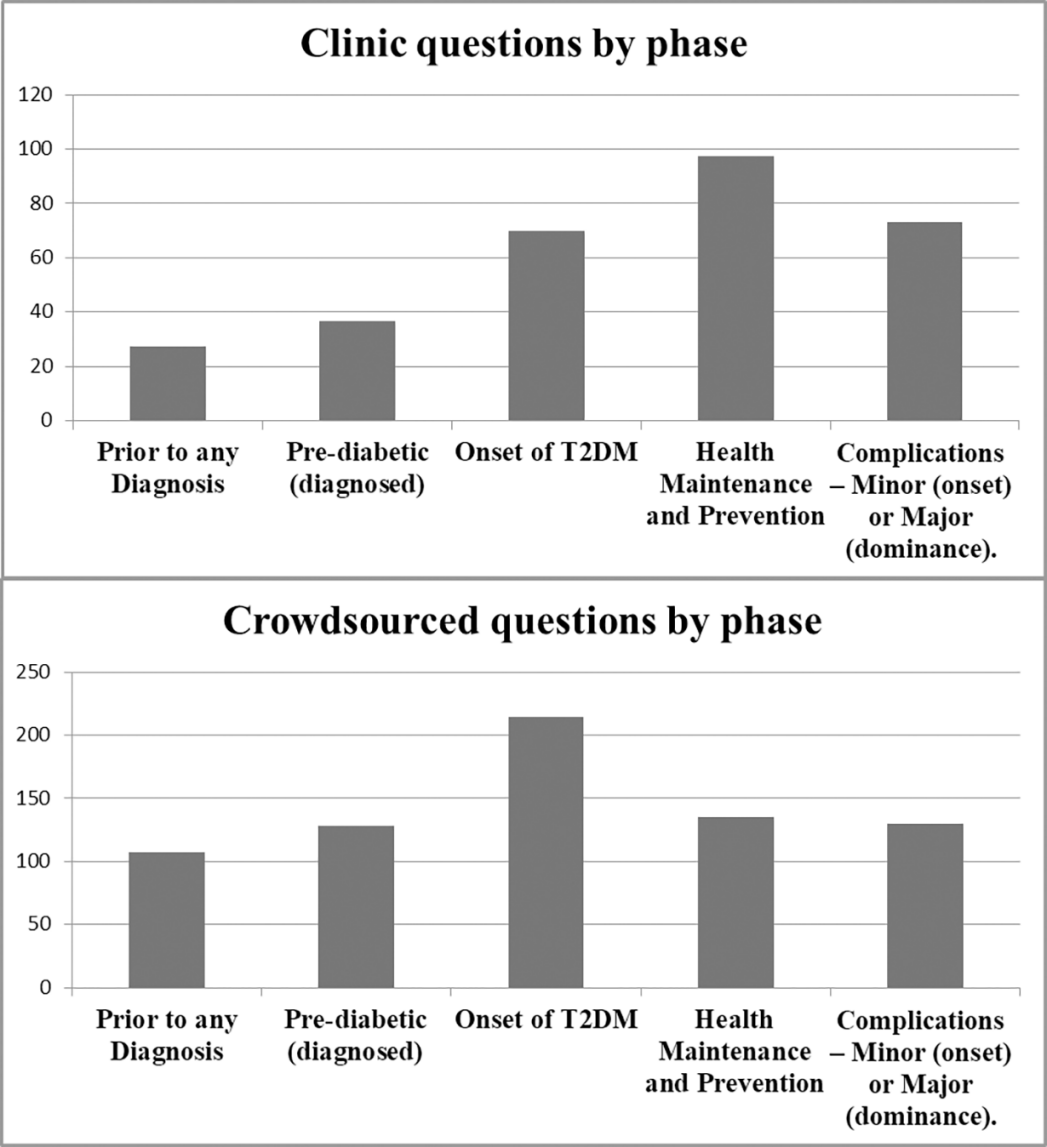
Clinic and crowdsourced questions by phase.

The most frequently applied phase in the clinic questions was Phase 4 (Health Maintenance and Prevention, 122 questions). This was followed by Phases 5 and 3, then Phases 2 and 1. The most frequently applied phase in the crowdsourced questions was Phase 3 (Onset of T2DM, 220 questions) followed by Phases 4 and 5, then Phases 2 and 1. Phase assignment numbers for clinic and crowdsourced questions are shown in Fig 1.

### Descriptive analysis

Our descriptive analysis identified four themes to pursue in future studies: (1) the specificity found in questions; (2) questions revealing the emotion behind an information need; (3) questions disclosing information needs not yet recognized in standard patient information resources; and (4) the potential for questions to identify specific constituent groups with their own information needs.

#### (1) The specificity of questions

Questions encourage specificity. The topic “diabetes and prognosis,” for instance, does not capture the specificity of the following four prognosis-related questions taken from our corpora:

- Is diabetes a death sentence?
- Will all Type 2 eventually go on to insulin?
- Is there any potential for a cure within the next few years, according to current research?

The first concerns a worst outcome, the second the inevitability of a treatment, and the third a best outcome. Numerous questions contemplated a decline in health, for example:

- Will it get worse [?]
- Will my condition only worsen [?]

Many asked about the likelihood and hoped-for outcome of specific treatments, for example:

- Can I ever reduce insulin & meds and feel good [?]
- Can diabetes be cured or rendered almost gone overtime through medicine and nutrition [?]
- Could a pancreas transplant cure diabetes in a person?
- What dictates the type of treatment needed/required for diabetes, and is directly injecting insulin ever avoidable?

Prognosis questions that were about a possible cure for diabetes were prominent. See S1 Table. All questions contained the word “cure,” or similar, such as “reverse,” “heal,” “[fully] go away,” “[completely] get over.” Many questions looked to scientific research for a cure and acknowledged it as a matter for the future. A smaller number specifically referred to diet or lifestyle changes, something an individual can do to affect the course of diabetes. Those asking such questions may be more receptive to taking action on their own behalf.

#### (2) Questions revealing the emotion behind an information need

Consider the following two questions, both ostensibly seeking to understand why the questioner has diabetes.

- How on earth I ever got diabetes in the first place. Never over weight blood pressure always fine never eat sweet food [?]
- Why me?

The first question shows some understanding of risk factors for the condition without, it seems, fully understanding genetic risks. Puzzlement and frustration are expressed. The second question is less a plea for information and more an expression of frustration and defeat. Its meaning, and what counts as an adequate answer, will differ depending on when it is asked–at diagnosis, at a periodic review of the patient’s care, when a new complicating factor has arisen that will affect self-management, or when there is a transition in care, such as with age-related changes or a change in the care team [77].

#### (3) Questions disclosing as yet unrecognized information needs

It is crucial that information on diabetes covers not only what health professionals consider important for people to know but also what the different constituent groups want to know, whether considered important to health professionals or not. Directly solicited, open-ended invitations to ask questions are a way to reveal information needs that may not be anticipated by health educators. Take the following questions that in effect ask for a severity index for diabetes.

- Are there variations in severity to diabetes and what determines severity?
- To what extent does it exist on a spectrum, such that people may be classified according to the degree to which they are diabetic, even if they are not diagnosable as diabetic according to present criteria?
- Are there variations in severity to diabetes and what determines severity?

This topic is covered in the literature [78] but not prominently or not at all in the trusted and vetted sources of patient-oriented diabetes information resources. It may be important to some patients’ needs to fully understand their condition. A related set of questions reveals a similar and important wish, whether feasible or not, to be able to monitor one’s health before it gets to a point of no return [79], as explicitly stated in this question:

- If you suspect you have Type 2 diabetes, at which point will it become impossible for you to reverse it by only changing your diet and exercise habits (and without requiring medication or the need to see a doctor?)

#### (4) Questions from specific constituent groups

Diabetes information is important not only for people with diabetes but also friends and family of people diagnosed with diabetes and for caregivers, those family members, neighbors, friends or paid persons who regularly look after someone with diabetes. Our corpora included several such questions.

- What are some ways to help a family member accept a diagnosis of diabetes?
- How hard is it to treat when the person who needs help isn't very receptive to their condition?
- What are some things you can do to help a family member better manage an appropriate diet for type 2 diabetes?
- What is the best way I can help my friends and family members with controlling their diabetes?

For other chronic conditions such as mental-health disorders, for instance, the role of family and friends is broadly acknowledged and discussed in education and information resources. Question collection on a massive scale may suggest a more prominent place for this topic in diabetes education.

## Discussion

### Principal results

The topics associated with the clinic questions (Own Health Record Related, Disease Complications, Treatment, and Psychosocial) confirm what might be expected, namely that patients whose condition is actively being managed are most concerned about complications of the condition specific to their medical history, with a primary concern being about psychosocial matters related to their disease. T2DM is a complex condition that has different disease progressions for different people and for the same person over time and as life circumstances change [69]. Significant effort has to go into making sense of the experience. A recent study comparing people seeking online health information for their own problem against those seeking information for someone else’s showed that the first group in contrast to the second focused primarily on symptoms and matters related to their own disease history [80].

The crowdsourced questions’ focus on Causes, Risk Factors. Prevention, Diagnosis, and Distribution of a Disease in a Population most likely reflects the fact that the crowdsourced questioners were in the main (91%) not themselves diagnosed but knew someone who was and so likely sought to understand what leads to diabetes and who among their family members may be at risk. Those seeking online information for someone else’s health problem have been shown to focus primarily on causes of a disease and disease terminology [80].

The stronger representation of Psychosocial questions from men warrants further investigation. Gender-based notions of masculinity have been shown for some people to be in conflict with effective self-management of T2DM, a central component in the treatment of diabetes [81]. The stronger representation of questions on Weight from women is perhaps not unexpected, but with recent research showing that men are developing T2DM at lower levels of adiposity than women, this may change [82].

The clinic questions, not surprisingly, predominantly concerned post-diagnosis issues whereas the onset of diabetes dominated crowdsourced questions. The number of crowdsourced questions asking, in effect, how you know if you have diabetes accounts for the high number of questions categorized under Onset of T2DM. Such a concern is consistent with the fact that over 30 percent of those with diabetes in the United States are unaware they have the disease [13]. It also perhaps indicates that the public health message about the prevalence of diabetes is being heard and people are wondering about their own health status.

### Related studies

There is a long and extensive record of questions being collected from health professionals and analyzed. Questions have been collected at the point of care, from email consultation with specialists, and through queries to information systems [26, 31, 33, 35–38]. Clinical questions have been categorized as to the kind of knowledge they sought and the kind of answers they needed, with taxonomic and other organizing structures proposed for them [24, 27, 35, 37]. The questions of family-medicine, elder-care, and rural-health physicians have been explored [25, 29, 30, 32, 34, 44]. Experiments have been done on different ways of capturing clinical questions through voice and other input media [28, 39–43]. Clinical questions associated with specific disorders have been evaluated, most notably cancer [42], and T2DM [31]. A systematic review of three decades of studies on clinical information needs [30] found that roughly 30% of the question types accounted for 80% of the questions clinicians asked, where a question’s type was relative to a 64-item taxonomy [24].

Studies of questions from healthcare consumers are relatively recent. In [45], 276 health-related questions posted on a social media question-answer website were subjected to qualitative content analysis, focusing on meta-characteristics of the questions such as the users’ motivations for asking the questions. In [46] and [47] a manual topic-based analyses of consumer questions was done using topics from the UMLS. In [49], 365 questions from a mailing list were analyzed in terms of topics and the type of question. In [50] and [51] smaller question collections (72 and 12) were subjected to detailed semantic, attitudinal or linguistic analysis. An increasing number of studies concern the development of question–answering technology for consumer health questions [83–87]. Patients have different information needs about T2DM at different points as their disease progresses. However little is known about these needs and how they change over time or across varying health or life circumstances [77]—even though there has been a significant amount of research on what the different phases of T2DM are [58–67]. It is in cancer care that the needs of patients at different stages of their disease have been most thoroughly studied [88–91]. These studies, show, for instance, that while most (91%) female breast-cancer patients wanted to know their prognosis before beginning adjuvant treatment [91], after the first consultation, their needs often shifted to matters of support, with 59–63% primarily wanting reassurance and hope and patients with advanced disease often desiring less information about their illness [88]. It is important that we develop a similar understanding of the changing needs of people with T2DM.

### Further research

The urgent need for resources allowing patients with T2DM to find answers to their questions has recently been documented [52]. One longer-term goal of this study is to develop a question-answer system, informed by the analysis of a very large number of questions and vetted answers and based on the automated identification of topics in questions. The twenty-three categories we devised for this study will almost certainly need further refinement, with a hierarchy of topics or an ontology possibly providing a better representation. In addition, *answer* topics as well as question topics need to be defined. For example, suppose a patient’s question is “I’m 44 and recently diagnosed with Type 2 diabetes, and now I am having difficulty reading fine print. Is this related to my diabetes?” This question falls into four possible answer categories. The first relates to temporary changes in eyesight when blood glucose fluctuates. The second concerns a side effect of the drug pioglitazone. The third is about diabetic retinopathy that leads to blindness. And the fourth concerns normal age-related changes in eyesight.

Finer-grained characteristics that are important in the management of diabetes are also needed. For example, the capacity of a person to act in any given environment (known as agency) seems to be expressed differentially in our questions [92]. The following question about a cure for diabetes appears to locate agency within the patient: “What stuff do you have to do to cure diabetes?” This is in contrast to a question that appears to locate agency within the broader society: “How close is science to finding a cure for diabetes?” If patients over time asked questions that differed in the location of agency, that would be of interest and possible clinical significance. In our follow-up studies when new questions are collected from patients, we will be labeling each question by the stage the questioner is in relative to his or her own disease progression. In this way a record of the questions asked in the aggregate by patients at each phase of the disease can be compiled along with the progression of questions for each patient individually, providing a broader and deeper perspective on the complex needs of those affected by or at risk of T2DM.

## Supporting information

S1 Table Questions about cure or reversal. (DOCX)

S1 File Clinic questions. (DOCX)

S2 File Crowdsourced questions. (DOCX)
