# Supplementary material for: Exploring patient information needs in type 2 diabetes: A cross sectional study of questions
Source: PLoS One. 2018 Nov 16;13(11):e0203429. doi: 10.1371/journal.pone.0203429 (PMC6239280; doi:10.1371/journal.pone.0203429)
Supplement: S1 Table — (DOCX) [file pone.0203429.s001.docx]

S1 Table. Questions about Cure or Reversal.

1. Is there a potential cure that is in the stages of early development?
2. How close is science to finding a cure for diabetes?
3. Right now diabetes is only manageable, is there anything on the horizon as far as a cure?
4. Could a pancreas transplant cure diabetes in a person?
5. Will diabetes ever be "cured"?
6. How likely is it that we will see a cure to diabetes in our life time?
7. Will stem cell research help cure diabetes?
8. Are researchers in any way hopeful that a cure is possible?
9. Is diabetes curable at all?
10. Will there ever be a time when diabetes is able to be reversed?
11. Information on developments in break throughs or cures. Transplants etc.^C^
12. Is there any cure in sight [?]^C^
13. Will there be a cure? ^C^
14. Is there any potential for a cure within the next few years, according to current research?
15. Is diabetes curable? As in, can you get completely over diabetes with no lingering effects? Or is it just something that you must live with?
16. Can diabetes be cured completely from a person who has it?
17. What are the chances of being cured for an adult male?
18. To find a cure and be able to take smaller doses of medication ^C^
19. Can Type II diabetes be “cured” or only managed [?]^C^
20. Can diabetes be cured?
21. Can Type II diabetes be cured completely?
22. Is diabetes ever curable?
23. If you have diabetes does it ever fully go away?
24. Since it seems to develop spontaneously, does it ever go away spontaneously?
25. Does it ever go away or leave the system [?]^C^
26. Make it go away ^C^
27. Can effects of TYPE 2 be reversed [?]^C^
28. Can diabetes be healed by changing your diet and eating whole foods vs processed foods?
29. Can Type II diabetes ever be reversed or cured, for instance, through correcting your diet and getting healthy?
30. Can type 2 diabetes symptoms be minimized, or fully cured, through lifestyle changes alone?
31. What stuff do you have to do to cure diabetes?
32. Once you have diabetes, can you live healthily enough to become cured of the disease's effects and essentially overturn it?
33. Can diabetes be cured or rendered almost gone overtime through medicine and nutrition [?]

Clinic questions are indexed by ^C^.

The last six questions (italicized) refer to actions a person can take on his or her own behalf
