# Supplementary material for: Exploring patient information needs in type 2 diabetes: A cross sectional study of questions
Source: PLoS One. 2018 Nov 16;13(11):e0203429. doi: 10.1371/journal.pone.0203429 (PMC6239280; doi:10.1371/journal.pone.0203429)
Supplement: S1 File — (DOCX) [file pone.0203429.s002.docx]

**S1 File. Clinic Questions.**

1. Could problem with balance be related to hypos? My GP thinks so and has reduced my gliclizide
2. Why has Victoza resulted in increased appetite and weight gain?
3. How do I stop need for sugar / chocolate?
4. How is my blood sugar controlled? Do I have to do anything more?
5. Why is there such a difference in blood tests?
6. My control has been good so far, but is it likely to deteriorate with age as my metabolism changes?
7. How do I obtain closer control of my blood sugar? I’m taking novomix 30
8. Still not taking drugs can I keep it under control with loss of weight?
9. Why do I have to take insulin?
10. Why are my tablets not working now?
11. How come I feel ok when my sugar levels reach 39 HI?
12. Would better diet help to get off insulin?
13. Why is my blood sugar up and down? And why can’t I manage it?
14. IS THERE ANY CURE IN SIGHT
15. DNAV HAS ANSWERED SO MANY OF MY QUESTIONS & PROVIDED A BETTER UNDERSTANDING OF DOSAGE. MY ONLY CONCERN IS ABOUT TIMES WHEN I HAVE INFECTIONS - TOO FREQUENTLY - WHICH ELEVATES THE BLOOD SUGAR = HIGHER DOSE -> HYPO
16. HOW TO REMAIN MORE POSITIVE ABOUT MY CONDITION. I GET FED UP CONSTANTLY FINGER PRICKING ETC - THEN I DO REALISE HOW LUCKY I AM TO HAVE ALL THE HELP I NEED!
17. I WOULD LIKE TO KNOW MORE INFORAMTION ON NEOROPATHY
18. OVER THE LAST 6 MONTHS I'VE FOUND THAT I'M BECOMING MORE AND MORE FORGETFUL. COULD THIS BE ATTRIBUTED TO MY DIABETES?
19. I'M CURRENTLY BEING INVESTIGATED FOR POSSIBLE SLEEP APNOEA. COULD THIS BE CONNECTED TO DIABETES?
20. WAITING ON DIETICIAN, SOME TIME NOW
21. IS THERE A DIABETIES GROUP TO TEACH WHAT FOODS SHOULD I MIX FOR BETTER CONTROL AS I HAVE REACHED STALEMATE FOR CONTROL
22. MY LEVELS HAVE BEEN HIGH FOR A LONG TIME CAN SIDE EFFECTS BE MADE BETTER AGAIN
23. WILL THERE BE A CURE?
24. CAN I GET AN IMPLANT THAT DOES YOUR INSULIN FOR YOU?
25. CAN GLAND START WORKING AGAIN
26. WHY DOES STRESS RAISE MY BLOOD SUGAR READINGS
27. CAN AN INSULIN PUMP HELP KEEP BETTER CONTROL THAN INSULIN PENS?
28. WHY DO MY HAND + FEET GET AFFECTED SO MUCH?
29. WILL I EVER STOP TAKING INSULIN AND TABLETS TOGETHER
30. WHAT ABOUT AN INSULIN PUMP
31. I AM TOLD I AM BORDERLINE HAVING TO TAKE INSULIN, I AM STILL UNSURE WHAT THIS MEANS AS MY BLOOD SUGAR IS GENARLY HIGH I THOUGHT INSULIN WAS FOR LOW BLOOD SUGAR
32. SOURCING INJECTION SITES - BRUISING
33. LFT RESULTS - ARE RAISED RESULTS DUE TO DIABETES?
34. HOW LONG HAVE I BEEN DIABETIC AND WHAT ARE THE LONG TERM IMPLICATIONS OF RAISED GLUCOSE LEVEL. CAN I START SOME TEATMENT TODAY?
35. HOW TO PREVENT ITS PROGRESSION! NEW THOUGHTS?
36. IF READING IS HIGH WHAT DO I DO? CAN'T GET A CONTANT READING ALWAYS UP & DOWN NEVER THE SAME.
37. COULD INSULIN BE AFFECTING MY SKIN? I HAVE AN ITCHY RASH AT PRESENT. MY SKIN IS VERY ALLERGIC TO VARIOUS PRODUCTS.
38. WHAT IS BRITTLE DIABETES
39. WHAT WILL HELP MY DIABETES
40. HOW TO MANAGE BLOOD SUGARS EASILY
41. QUICKER ACTING TREATMENT
42. MY BLOOD SUGARS ARE RISING EVEN THOUGH I HAVE INCREASED MY INSULIN INTAKE ACCORDINGLY. MY FOD INTAKE HAS NOT RISEN TO EXPLAIN THIS WHICH IS A WORRY
43. WILL IT AFFECT MY WORK
44. IN FUTURE, IS THERE ANY LIKELIHOOD OF DIFFERENT TREATMENT OTHER THAN INJECTION E.G. INSULIN PATCHES
45. WHY IS IT GETTING HARDER FOR ME TO MAINTAIN CONTROL OF MY BLOOD SUGARS AS I GET OLDER?
46. DOES IT EVER GO AWAY OF LEAVE THE SYSTEM
47. CAN I DO MORE TO HELP MYSELF
48. IMPACT OF ALL MEDICATIONS ON PERIODIC BLOOD TEST RESULTS AND THE EFFECT ON MY DIABETES CONTROL.
49. PERIODIC ASSESSMENT / EXAMINATION WITH DOCTORS AT KEY MILESTONES.
50. HOW IS RESEARCH GOING
51. MORE INFORMATION ON DIET
52. EFFECTS ON MY EYES
53. GENETIC IMPLICATIONS WITH MY FAMILY
54. METFORMIN - AM I TAKING TO MANY TABLETS I TAKE 4 GLICLAZIDE PER DAY AND 2 METFORMIN
55. THE PREVIOUS RISE IN MY BLOOD SUGAR COUNT WAS MY HIGH INTAKE IN BANANAS. I WAS UNAWARE OF THEIR HIGH SUGAR CONTENT - 2-3 PER DAY
56. ARE THERE ANY NEW TREATMENTS BECOMING AVAILABLE
57. IS LIFESTYLE OR DIET THE BIGGEST FACTOR IN HELPING TREATMENT
58. HELP WITH WEIGHT ISSUES
59. INSULIN PUMPS
60. WHY MY BLOOD SUGARS ARE STILL HIGH DESPITE BIET AND DRUGS?
61. WILL IT GET WORSE
62. CAN MEDICATION EVENTUALLY REPLACE INJECTION
63. MY EXTENDED FAMILY - SHOULD THEY BE REGULARLY TESTED
64. NOT ENOUGH INFO FOR THING'S YOU CAN EAT
65. IS IT UNDER CONTROL?
66. WEIGHT IS NOT MOVING.
67. CONCERN ABOUT MEDICATION.
68. DO I REALLY NEED IT NOW
69. GETTING INSULIN PUMPED IN AS YOU NEED IT RATHER THAT HAVING TO INJECT ALL THE TIME.
70. MORE INFORMATION ON DIET AND HOW IT AFFECTS DIABETES?
71. HOW OTHER MEDICATIONS INTERACT WITH DIABETES AND DIABETIC MEDICATION
72. THE BALANCE BETWEEN WHAT IS EATEN & WHAT DOSE OF INSULIN COUNTERACTS
73. HOW TO BALANCE FOOD, ACTIVITY AGAINST INSULIN ESPECIALLY WHEN WORK ACTIVITY IS NOT A CONSTANT
74. WHAT IS AN ACCEPTABLE HBA1C AND DOES THIS VARY FROM PATIENT TO PATIENT?
75. WHAT ARE THE MAJOR ADVANCES IN CARE AND WHEN ARE THE BENEFITS OF THESE LIKELY TO BE AVAILABLE TO PATIENTS?
76. HAVING TIREDNESS ALL THE TIME
77. TIMES FEELING WEAK
78. WHAT ARE THE SERIOUS SIDE EFFECTS OF DIABETIC MEDICATION?
79. WHAT LONG TERM DAMAGE CAN THE SIDE EFFECTS OF MEDICATION CAUSE?
80. WHY I CANT GET MY SUGARS DOWN
81. I WOULD LIKE TO KNOW WHY IT TOOK SO LONG TO BE FOUND THAT I HAVE TYPE 2 DIABETES BECAUSE FOR YEARS DOCTORS KEPT ASKING ME IF I WAS DIABETIC
82. WHAT IS MYOPTHY AND IS THERE ANYTHING THAT I CAN DO TO HELP WITH IT
83. WILL MY CONDITION ONLY WORSEN
84. IS THERE AN ALTERNATIVE TO INSULIN
85. AM I TAKING THE CORRECT DOSE?
86. CAN I TAKE MY "BACKGROUND" AT A DIFFERENT TIME OF DAY TO GET BETTER CONTROL
87. WHY DOES MY URINE HAVE ABNORMALITIES IN IT?
88. WHY DO BLOOD LEVELS VARY
89. IS UNSTEADINESS WITH LEG MOVEMENTS DUE TO DIABETES
90. HOW IT AFFECTS MY LIFE EXPECTANCY
91. ITS EFFECTS ON MY EYESIGHT
92. WHY DO DOCTORS NOT FULLY UNDERSTAND BERTIG
93. ARE THERE ANY EASIER WAYS OF MONITERING BLOOD SUGERS?
94. HOW TO REALLY REDUCE MY BACKGROUND INSULIN - EXERCISE, DIET,WEIGHT LOSS
95. INSULIN RESISTANCE, ABSORPTION, ETC.
96. NEED TO ROTATE SITES MORE FREQUENTLY
97. CAN I EVER REDUCE INSULIN & MEDS AND FEEL GOOD.
98. I WOULD LIKE TO BE MORE CONFIDENT ABOUT MY DIABETES - HOW CAN I ACHIEVE THIS.
99. ANY NEW INSULINS
100. PUMP EDUCATION
101. INFORMATION ON DEVELOPMENTS IN BREAK THROUGHS OR CURES. TRANSPLANTS ETC.
102. INFORMATION ON POSSIB ILITY OF CHILDREN GETTING DIABETES, INHERTITED?
103. HOW ON EARTH I EVER GOT DIABETES IN THE FIRST PLACE. NEVER OVER WEIGHT BLOOD PRESSURE ALWAYS FINE NEVER EAT SWEET FOOD
104. HOW THINGS WILL PROGRESS IN THE FUTURE
105. IS THERE ANY POINT IN SEEING ME EVERY 10 MONTHS.
106. WHY ME?
107. MAKE IT GO AWAY
108. THE RISK OF LOSING A LIMB OR DAMAGE TO ORGANS
109. TO FIND A CURE AND BE ABLE TO TAKE SMALLER DOSES OF MEDICATION
110. INFO RE DIABETES & PREGNANCY
111. WILL SIGHT DETERIORIATE MORE RAPIDLY HAVING DIABETES.
112. WILL ALL TYPE 2 EVENTUALLY GO ON TO INSULIN?
113. WHY WHEN WATCHING WHAT YOU EAT YOUR BLOOD SUGAR STILL GOES UP A LOT
114. IT HAS INFECTED SKIN IS THERE ANYTHING CAN BE USED TO HELP THIS AS SKIN BREAKS OUT IN SPOTS AND BLEEDS.
115. CAN EFFECTS OF TYPE 2 BE REVERSED
116. WILL I HAVE TO END UP ON INSULIN WHEN IT ADDS WEIGHT TO MY ALREADY HEAVY BODY?
117. CAN TYPE II DIABETES BE "CURED" OR ONLY MANAGED
118. HOW DOES AGEING OR OTHER ILLNESS IMPACT ON SOMEONE WITH DIABETES
119. WHY I CONTINUE TO GO TO THE BATHROOM EVERY HOUR
120. WHY I AM ALWAYS TIRED
121. WILL IT PASS ON TO MY CHILDREN?
122. FEET SEEN TO EVERY FOUR MONTHS IT USED TO BE EVERY THREE MONTHS. WHY WAS IT CHANGED?
123. I LAST HAD MY FEET SEEN TO TWO MONTHS AGO, MY TOE NAILS ARE ALREADY LONG AND GIVING ME SOME PAIN
124. FEET + FINGERS
125. HOW I AM DOING.
126. AND MY FEET.
127. AM I EATING THE RIGHT THINGS
128. HOW WELL AM I MANAGING MY DIABETES? ARE THE DECISIONS I AM TAKING KEEPING IT UNDER CONTROL OR AM I DETERIORATING?
129. IS THERE A LIFE SPAN TO HAVING DIABETES?
130. HOW OFTEN SHOULD I DO MY BLOOD SUGAR LEVEL
131. MY WEIGHT GAIN OVER LAST YEAR
132. PASSAGE OF INFORMATION KEPT INFORMED
133. COMMON COMPLICATIONS?
134. BLOOD RESULTS & WOULD LIKE TO KNOW IS THER ANY IMPROVEMENT FROM PREVIOUS & AT PRESENT?
135. POTENTIAL TRAILS AVAILABLE TO TRY INTO EASING REGULAR AND DAILY TREATMENT
136. THE DIABETIC PROGRAMS "BERTIE" ETC WHY CANT THEY BE AVAILABLE ONLINE TO HELP PEOPLE WITH BUSY SCHEDULES WHO CANT ATTEND RECEIVE THIS PROGRAM
137. WHEN MY BLOOD SUGARS ARE HIGH TO BRING THEM DOWN SHOULD I TAKE SOME INSULINE AT THIS STAGE.
138. IS SUGAR STRICTLY NOT, ALLOW OR IS IT ALLOWED IN MODERATION
139. UNDERSTANDING CARBOHYDRATE INTAKE AGAINST INSULIN & IT NOT BEING THE SAME RESULTS EACH TIME?
140. KNOWING IF THE MEDICATION I HAVE BEEN BEING PERSCRIBED IS THE CORRECT STUFF FOR ME??
141. LONG TERM CARE OF MY CONDISHION IF I WAS TO BECOME BLIND
142. HOW LONG BEFORE YOU FIND A TREATMENT THAT WILL GIVE BETTER CONTROL OF BLOOD GLUCOSE LEVELS
143. WHY DO SOME PATIENTS NEED AMPUTATIONS
144. WOULD LOVE TO HAVE A BOOK OF REFERENCE OF DO'S & DON'TS WITH REGARDS TO FOOD
145. WHY WHEN I DON'T EAT DOES MY SUGAR GO UP?
146. I DON'T KNOW WHAT TO EAT AS I HAD OTHER SURGERY WHICH STOPS ME EATING FRUIT & VEG
147. GETS ME DOWN SO MUCH.
148. HAVING BEEN TYPE 1 FOR 30 YEARS AS I GET OLDER I GET STRESSED MORE EASILY AND FOR MORE PROLONGED PERIODS. IS THIS RELEVENT TO MY DIABETES OR JUST SOMETHING THAT COMES WITH AGE?
149. PERIPHERAL NEUROPATHY PLAGUES ME CONSTANTLY. MEDICATION SUBDUES IT BUT ARE THERE ANY OTHER TECHNIQUES I CAN USE TO EASE THE PAIN?
150. DO DIABETES CAUSE CRAMPS IN ONE'S LEGS, OR IS IT THE TABLETS.
151. I WOULD LIKE TO NO IS THERE ANY OTHER PARTS OFF MY BODY I CAN INJECT MYSELF
152. THE COMPLICATIONS THAT DIABETES CAN CAUSE (IE HEART FAILURE)
