# Supplementary material for: Exploring patient information needs in type 2 diabetes: A cross sectional study of questions
Source: PLoS One. 2018 Nov 16;13(11):e0203429. doi: 10.1371/journal.pone.0203429 (PMC6239280; doi:10.1371/journal.pone.0203429)
Supplement: S2 File — (DOCX) [file pone.0203429.s003.docx]

**S2 File. Crowdsourced Questions.**

1. Under what circumstances do those with diabetes need to give themselves an insulin injection?
2. Is diabetes something that can be overcome or when someone has it, do they have it for life?
3. What kind of special diet does someone with diabetes need to follow and are there any foods they cannot have at all?
4. Under what circumstances is a ketogenic diet an effective treatment?
5. To what extent does it exist on a spectrum, such that people may be classified according to the degree to which they are diabetic, even if they are not diagnosable as diabetic according to present criteria?
6. Given the role of mitochondrial dysfunction in the pathogenesis of diabetes, what relation might the disease have to other diseases involving mitochondrial dysfunction, such as cancer?
7. What are some early symptoms related to diabetes
8. How can one contract diabetes so late in life?
9. Does eating a lot of sweets cause diabetes?
10. What are foods besides candy and other high sugar foods that diabetics should avoid?
11. I have heard the phrase 1.5 diabetes, and would like to know it's basis
12. On what part of their body do diabetics inject insulin?
13. Does cholesterol play a factor in the diagnoses of diabetes?
14. Fat acceptance advocates state that "most overweight people never develop type 2 diabetes", so if this is true, why is Type 2 diabetes considered a fat person disease?
15. If you suspect you have Type 2 diabetes, at which point will it become impossible for you to reverse it by only changing your diet and exercise habits (and without requiring medication or the need to see a doctor?)
16. What is it about being obese that makes one more prone to Type 2 diabetes - what's different about an obese body that invites this type of diabetes?
17. How much will you have to change your lifestyle to deal with diabetes?
18. What will your diet be like when dealing with diabetes?
19. Do you ever get over having to prick your finger in order to test your blood sugar?
20. What is the most painful aspect of having diabetes?
21. How does diabetes affect a person's daily life and activities?
22. As a person ages, does diabetes become more troublesome and harder to treat?
23. I understand that two fasting readings over 126 triggers a diagnosis of diabetes II, but will a doctor automatically put a patient on medication at this point, or still give the patient a chance to bring those levels down with diet and/or exercise?
24. I have read that Type II diabetes can sometimes be reversed with a substantial change in diet and exercise habits. Is this true?
25. If I have been diagnosed with pre-diabetes, can I prevent full-blown diabetes if I get serious about good eating habits and exercise?
26. What can we as a community do to address and stop the spread of diabetes?
27. What sorts of lifestyle changes should a person make that has recently been diagnosed with diabetes?
28. Can diabetes be prevented at a young age?
29. What are ways to tell if you have diabetes? (Such as symptoms someone experiences)
30. Is "Adult Onset Diabetes" a real thing, can you get diabetes from just gaining too much weight?
31. If Adult Onset Diabetes is a real thing can you get rid of diabetes by doing things such as losing weight?
32. What are the first symptoms of diabetes?
33. What is the biggest step one can take to prevent ever getting diabetes?
34. Does a person with diabetes have a shorter life span than those without diabetes?
35. Can type 2 diabetes symptoms be minimized, or fully cured, through lifestyle changes alone?
36. How can you manage genetic risk factors for type 2 diabetes?
37. What are the most important lifestyle changes for a type 2 diabetic to pursue?
38. My mother experienced complete liver failure not too long ago, and was diagnosed with Type II diabetes about 10 years ago. Could liver failure have been caused by diabetes?
39. Is it likely that Type II diabetes could really be Type I diabetes that went undiagnosed for decades?
40. Can Type II diabetes be cured completely?
41. Does having diabetes mean that I am at higher risk for other medical problems?
42. How often should I test my blood sugar, and what should I do if it is too high or too low?
43. Are there any new medications that I could use to help manage my diabetes?
44. Does diabetes always lead to limb complication with progressing age?
45. Does diabetes directly cause, or increase the risk of other serious diseases?
46. Is there a genetic pre-disposition to diabetes? Or is it purely based on diet?
47. Will diabetes ever be "cured"?
48. Why aren't doctors more proactive in helping patients avoid the other issues that may form over time, such as depression, neuropathy, etc?
49. Why do insurance companies fight so hard against new treatments for diabetics?
50. Are there any natural remedies for diabetes that have shown any promise at all? I understand that no natural remedy is FDA approved, but that doesn't mean that they don't work
51. Is diabetes curable? As in, can you get completely over diabetes with no lingering effects? Or is it just something that you must live with?
52. Are there ways to know if you are susceptible to diabetes (I know it is passed down from family members) even if you don't know if your family has had a history of it?
53. What are the best ways to prevent getting diabetes?
54. Are there variations in severity to diabetes and what determines severity?
55. Is diabetes genetic and what is the likelihood of a child getting it if a parent has it?
56. What are the major differences in severity and symptoms of type 1 and type 2 diabetes?
57. What can I do to prevent contracting either type of diabetes and what symptoms should I look out for?
58. What are the treatments available to those with diabetes, and how do they help improve the quality of life?
59. Can regular exercise be helpful in preventing the onset of diabetes?
60. What specific kinds of foods are likely to contribute to the onset of diabetes?
61. Are there any natural herbs that are useful in treating diabetes?
62. Is diabetes a death sentence?
63. Can you still eat sugary things with diabetes?
64. Can it be controlled naturally or you have to take medication?
65. What are good ways to track the onset of diabetic complications in a person?
66. What are some ways to help a family member accept a diagnosis of diabetes?
67. What is the biggest cause of getting diabetes - health/weight related or family history related?
68. How much does it shorten someones lifespan if they're able to keep it in check?
69. How hard is it to treat when the person who needs help isn't very receptive to their condition?
70. What are the consequences of not treating it like you should?
71. Does drinking water help to prevent diabetes?
72. How much sugar can the average person intake without having to worry about diabetes?
73. Why do limbs sometimes have to be amputated for diabetes?
74. What is the biological mechanism that causes diabetes?
75. Is there any potential for a cure within the next few years, according to current research?
76. Is it more important to regulate insulin uptake, or focus on diet based solutions?
77. What causes diabetes?
78. What stuff do you have to do to cure diabetes?
79. How does the medicine cost for diabetes?
80. what organ does it affect?
81. how does it manifest?
82. who is likely to get diabetes?
83. What are the main differences in symptoms between type 1 and type 2 diabetes?
84. How much money per year does diabetes cost the federal government due to health costs?
85. Is there a potential cure that is in the stages of early development?
86. Is diabetes hereditary? Or is it mainly caused by poor lifestyle choices?
87. Can you have diabetes and not realize it?
88. Can you ever heal yourself of diabetes? To the point where you have no symptoms?
89. What can I do to prevent the onset of diabetes as I age? Is is preventable based on diet and lifestyle, or inherently inevitable?
90. Child onset diabetes seems to be growing. Why are more children being effected?
91. Why does it seem like their are more individuals who are being severely negatively effected by diabetes- lose of limbs, eyesight, etc?
92. What is the life expectancy with someone that has diabetes ?
93. What is the oldest a person could be when they are diagnosed with diabetes ?
94. Is it more common for male or females to be diagnosed ?
95. Why does it seem that most (if not all) charities I see geared towards fundrasing for diabetes is focused on type 2 only?
96. How much sugar can one have on a regular basis before having to worry bout diabetes?
97. Can anyone get diabetes or is it in your genes
98. if you have diabetes does it ever fully go away?
99. What are some of the newest methods of treating diabetes?
100. How limited of a diet does a person with diabetes have, or what are some main food types that they should avoid?
101. How close is science to finding a cure for diabetes?
102. What improvements in artificial insulin do doctors and researchers expect in the next 5-10 years? How will such improvements and advances help diabetics control their blood sugars?
103. is it deadly?
104. how can I manage diabetes?
105. Can I get rid of diabetes thru exervise and diet?
106. Can a Diabetic eat as many carbs as they want as long as they have insulin?
107. What happens if Diabetes is left untreated and how long can you live untreated?
108. What are some things you can do to help a family member better manage an appropriate diet for type 2 diabetes?
109. What exactly is the difference between type 1 and type 2 diabetes?
110. Right now diabetes is only manageable, is there anything on the horizon as far as a cure?
111. Is diabetes reversible and, if so, how is it possible?
112. What are the common symptoms of diabetes and how do you know when to seek medical attention?
113. How is "prediabetes" distinct from diabetes?
114. When you are diabetic can putting lotion on your feet cause sores?
115. What are simple ways in which you can eliminate temptation and stay away from high sugar foods?
116. Are my children or grandchildren more likely to be diabetic and when should they begin testing, when are they at risk?
117. What are the differences between type 1 and type 2?
118. If someone gets diabetes, does he always have it or can it disappear?
119. Can babies be born with diabetes?
120. How does diabetes lead to someone having to amputate their limb?
121. Is it only obese people who get diabetes?
122. Why does having diabetes sometimes cause someone to become blind?
123. What is the best long term options for keeping type 2 diabetes in check?
124. What is the best way I can help my friends and family members with controlling their diabetes?
125. What lifestyle changes can I make to make sure that I don't end up contracting type 2 diabetes when I get older?
126. What should I do with someone who is hypoglycemic?
127. If someone is so hypoglycemic they are unconscious, what can I do to get them to regain consciousness?
128. What are the early warning signs someone is hypoglycemic?
129. what are the best foods to eat regularly when you have diabetes?
130. what foods should one avoid when they have diabetes?
131. is becoming a vegetarian helpful in controlling diabetes?
132. What is the science behind diabetes that causes ones immune system to attack itself?
133. When it comes to children fighting diabetes, what is the youngest age they should be allowed to give themselves injections?
134. I know that exercise can be good for diabetes, but are there any specific exercises that have stronger results?
135. Can eating a low carb diet turn type 2 diabetes around?
136. Will pre-diabetes always turn into diabetes?
137. Is type 2 diabetes hereditary or dietarily related?
138. Is diabetes a genetic condition that makes some people more prone to contracting it?
139. What can be done to prevent diabetes and are preventative measures effective?
140. How has treatment for diabetes advanced in recent years?
141. Could a pancreas transplant cure diabetes in a person?
142. Can diet changes stave off onset of diabetes in someone genetically predisposed to get it?
143. What part does increased body fat play in the the increased risk of diabetes?
144. What is the average weight of a person who is diagnosed with type 2 diabetes?
145. What kinds of foods should people with type 2 diabetes stay away from?
146. What are some preventative steps to take if your family is known to contract diabetes ?
147. If I contract diabetes, is it true that I can combat it with a very excellent diet instead of taking medication ?
148. Is there surgery that can be done to 'fix' the cause of diabetes (that is, fix the gland that is responsible for sugar control)
149. Is there any one symptom or group of symptoms that a person might experience early on that forecasts the onset of diabetes?
150. How has the daily maintenance and checking of blood and administering medication changed over the last 10 years for diabetics?
151. How much risk does a slightly overweight person face with regards to the onset of diabetes later in life, and will better nutrition prevent it?
152. What is the life expectancy of someone with type 2 diabetes?
153. My mother has type 2 diabetes. Does that raise my risk?
154. Can diabetes affect a person mood?
155. What are some of the early symptoms of diabetes?
156. Can Type II diabetes ever be reversed or cured, for instance, through correcting your diet and getting healthy?
157. Have any new drugs to treat diabetes been approved in the past few years?
158. How much damage does injecting yourself with insulin do over the course of say 5 or 10 years and what are the signs something his happening internally (eg kidneys, pancreas, other vital organs, etc.
159. I've seen digital pumps you can wear. Are there any market solutions that take in that feedback and give you recommended actions to keep blood sugar levels lower?
160. Are there any market solutions for inputting food values and giving you a projected blood sugar level after consuming food?
161. What kind of diet changes are most effective in preventing or aiding in treatment of diabetes?
162. If you had been obese and loose weight, does your risk for diabetes lower significantly, even if other members of your family have diabetes?
163. Are there warning signs you should be on the look out for if you have a significant risk of diabetes and what are they?
164. What sort of diet should I eat to ensure that I do not develop diabetes?
165. What types of diabetes are genetic and which are caused by lifestyle choices?
166. What types of individuals are most at risk of developing diabetes?
167. Will diabetes cause more health issues down the road such as organ failure, cancer or other diseases?
168. Do any races have a higher chance then others of getting diabetes?
169. Are the symptoms for diabetes the same for both type 1 and type 2 and are they hard to spot?
170. What are some of the very subtle and early signs that you are developing diabetes, some of the very first things you experienced that you maybe might have thought was caused by something else, perhaps?
171. How different do you feel on days when you are managing your diabetes well versus days that you are not, and how much of an effect does that have on your life?
172. Do you personally think you will ever make the lifestyle changes necessary to completely get off of any kind of medicine you are on, or at least the ones that that might apply to?
173. How does it affect you day to day?
174. Is sugar completely forbidden or are you allowed a moderate amount occasionally?
175. Are there unique troubles or situations that you are more aware of than people not afflicted?
176. how can i lose a leg from this?
177. how will this effect my mobility
178. what does my family need to know?
179. How likely is it to get diabetes if somebody in your family has it?
180. Can somebody be born with diabetes if the mother has it, or is it something that only happens due to environment or behavior?
181. Which type of diabetes is worse to have health-wise?
182. How long can I live with diabetes if I do all my medication on time?
183. How often do I need to check my blood sugar for optimum health?
184. It it possible to leave insulin in the car on a warm day and will it hurt the medication?
185. How does the usual diet for a person with diabetes look like?
186. Are there regular symptoms for people who might not know they have diabetes?
187. How invasive is the diabetes test, and how much does it normally cost?
188. I am curious as to the actual genetic inheritance risks with diabetes - In particular, how likely is someone with a family history of diabetes (yet not having the disease themselves) to have a child with a high predisposition for the disease?
189. How often should someone with a predisposition for diabetes be tested?
190. If someone has a family history of diabetes, what extra steps can they take in their diet/exercise habits to prevent the possible onset of the disease?
191. Are diet soft drinks really better for diabetics than regular soft drinks?
192. What is a misconception that most non-diabetics would have in regards to living with diabetes?
193. What dictates the type of treatment needed/required for diabetes, and is directly injecting insulin ever avoidable?
194. How often is testing your blood sugar levels required, and is it invasive to everyday life?
195. Is diabetes curable at all? Will there ever be a time when diabetes is able to be reversed?
196. Does diabetes actually hurt when you have it? Outside of numbness of extremities, do you actually have other body pains?
197. What is the earliest age you can contract diabetes?
198. How likely is it that we will see a cure to diabetes in our life time? Will stem cell research help cure diabetes?
199. There seems to be an increase in people who have diabetes in the United States, is this because we have better testing tools or is it because Americans are now living unhealthier lives than before? Is it something else entirely?
200. I have noticed that some people like my parents can take a pill of metformin to control diabetes but others like my grandmother needs to take a shot of insulin. Is this how the disease progresses or are these two treatments not related? How likely is it that someone would need to start taking insulin shots as they get older?
201. Do you have to take insulin from a pig?
202. Does it affect your psychological state or is it just physical?
203. Are researchers in any way hopeful that a cure is possible?
204. Is diabetes hereditary or do certain foods cause it?
205. Can diabetes be cured completely from a person who has it?
206. Once a person is diagnosed with diabetes, what can they do to keep a balanced lifestyle?
207. Is there strong evidence that diabetes is genetic or is it more of a lifestyle disease?
208. Can diabetes be healed by changing your diet and eating whole foods vs processed foods?
209. How many people are expected to be affected by diabetes in the next 20 years?
210. What is the average life span of someone living with type 2 diabetes?
211. If my partner were to get diabetes of either type, would I be at risk of catching it through unprotected sex?
212. If I had children with my diabetic partner, what is the likelihood our child would eventually get diabetes?
213. Does loss of extremities occur commonly in diabetes sufferers or is that somewhat rare?
214. Once you have diabetes, can you live healthily enough to become cured of the disease's effects and essentially overturn it?
215. Do people with diabetes that is under control have shorter average lifespans than non-diabetics?
216. How much does diabetes affect your vision?
217. Can diabetes be something that a healthy person gets?
218. How can a healthy person lower their chances of getting diabetes?
219. What should i be telling a child's new school about how to manage my kids type 1 diabetes?
220. When my child's goes to have play dates, what candy's and sweet's should I tell the parents not to give my kid, and which kinds are ok for my kid to have?
221. Are there certain type of exercises my child can not do? Also what are some that my child can do and still be able to manger his diabetes?
222. Can diabetes be cured?
223. Is it harder for slender people to have diabetes?
224. Is it easier to have diabetes when a person is over forty years old?
225. Can simply eating too much sugar cause diabetes in a healthy person?
226. Is diabetes mostly genetic or is it caused by external factors?
227. How much of a role does diet play in preventing diabetes?
228. Are drugs the best way to control diabetes?
229. Once Type II Diabetes has set in, can it be reversed?
230. What about diabetes (blood sugar) causes you to lose limbs or eyesight?
231. What are the pros and cons of the different methods of insulin injection?
232. What is the relationship between carbohydrates and diabetes?
233. What is being done on a national level to help reduce the incidence of diabetes in children?
234. What is the difference between type 1 and type 2 diabetes?
235. Is it true that low carbohydrate diets can reverse type 2 diabetes?
236. Do people with controlled diabetes have a shorter life expectancy than normal?
237. What changes have you needed to make to your diet since being diagnosed with diabetes?
238. What was your A1C score when you were diagnosed, and what is it currently at your point in treatment?
239. Have you begun an exercise regimen in order to control your blood sugar levels?
240. Other than my doctor testing me, are there early signs and symptoms of diabetes that I should look out for?
241. How effective are diabetes medications
242. When is it most likely to happen to an adult male?
243. Which type of diabetes do you have to inject insulin?
244. What are the chances of being cured for an adult male?
245. What is the average lifespan of somebody diagnosed with diabetes?
246. Why is diabetes called the "silent killer"?
247. What is the best natural treatment for diabetes, not a prescription, just a natural remedy?
248. Are there other methods of administering insulin in children that is less painful and invasive than shots?
249. When will the artificial pancreas be available?
250. How has stem cell implantation worked?
251. What is the average lifespan of a person after diabetes diagnosis?
252. How painful is injecting insulin and how often people do it?
253. How costly is regular treatment for diabetes without insurance?
254. Is it true you can lose a foot?
255. CAn eating fast food cause me to g et it?
256. If I consume too much sugar am I more liable to come down with it?
257. Why do some people with type two need Insulin and some do not?
258. What happens if a diabetic keeps eating sugary foods, what are the long term problems caused by diabetes?
259. If you start taking insulin but lose weight and eat right can you eventually come off of the Insulin?
260. How likely is it that a diabetic will require an amputation at any point in their lives?
261. How much higher is the risk for a smoker to contract diabetes compared to non-smokers?
262. What are the best ways that a diabetic can prevent complications like strokes or blindness?
263. Can disabetes be cured or rendered almost gone overtime through medicine and nutrition
264. How expensive are diabetes treatments over time
265. How early can a person develop diabetes
266. How does diabetes affect your ability to have sexual intercourse and reproduce?
267. Is it possible for you to have type 1 diabetes and then get type 2 diabetes (or vice versa)?
268. Is weight gain the only way to get adult diabetes?
269. Why do certain children have diabetes?
270. Are all people as equally prone to diabetes?
271. My father had Type 2 diabetes, and I would like to know if that increases my chances of getting it?
272. What can I do to improve my health to avoid getting it, things like diet choices
273. What is the difference between Type 1 and Type 2 diabetes?
274. What type of diet should a diabetic follow?
275. Can symptoms of diabetes lead to pancreatic cancer?
276. What are the essential differences between diabetes types?
277. Is there variation in the severity of individual diabetes for both types?
278. Is diabetes ever curable?
279. would checking my blood sugar level periodically using a retail checking device be suitable in helping to prevent diabetes?
280. i understand i can be predisposed to diabetes based on my pedigree, but if only my great grandparent had diabetes, would it mean i could possibly get it genetically?
281. would taking both a pill and insulin shots help ward off the symptoms of diabetes or would they both counterract each other and provide no benefit?
282. wha does diabetes do that causes the high pressure in the eyes that causes loss of eyesight?
283. How does diabetes affect the circulation which can cause loss of limbs?
284. Since it seems to develop spontaneously, does it ever go away spontaneously?
